# Dilemmas of Ethics in Practice in Longitudinal Health Research: Identifying Opportunities for Widening Participation of Residents

**DOI:** 10.3389/fsoc.2019.00033

**Published:** 2019-04-25

**Authors:** Rhian Twine, Gillian Lewando Hundt, Kathleen Kahn

**Affiliations:** ^1^MRC/Wits Rural Public Health and Health Transitions Research Unit (Agincourt), School of Public Health, Faculty of Health Sciences, University of the Witwatersrand, Johannesburg, South Africa; ^2^Division of Health Sciences, Warwick Medical School, University of Warwick, Coventry, United Kingdom; ^3^INDEPTH Network, Accra, Ghana; ^4^Epidemiology and Global Health Unit, Department of Public Health and Clinical Medicine, Umeå University, Umeå, Sweden

**Keywords:** ethics in practice, longitudinal health research, widening participation, informed consent, returning individual results

## Abstract

**Background:** Mechanisms for widening participation of local participants in research studies can improve governance of public health research. Research conducted in longitudinal health study areas depends on there being mutual trust and respect over time between the local residents and researchers. Ethics in practice needs consideration alongside procedural ethics. By widening participation of the experimental public—local residents and resident service providers—ethics in practice and accountability are strengthened.

**Methods:** The study was undertaken in a longitudinal health study area in rural South Africa using multiple qualitative methods. The sample included 35 individual and five group interviews with resident local leaders and service providers, 24 individual and eight group interviews with residents of the study area, and ten researchers' reflections on two critical incidents from ethnographic field notes on dilemmas of ethics in practice. The interviews were all audio-recorded (besides one where consent to record was not given) and then transcribed verbatim and translated from Shangaan into English. Thematic analysis was conducted.

**Results:** Residents requested the reporting back of personal screening test results from research studies, and raised informed consent issues. Researchers recognized the importance of mechanisms to increase their accountability to residents throughout the research process, and the complexity of informed consent and fieldwork procedures within research studies.

**Conclusion:** This study elicited the views of residents and researchers in a longitudinal health study area to seek guidance on how to strengthen participation in research governance. Three strategies were identified by participants to widen participation of the experimental public. Firstly, increasing study budgets so that individual screening test results could be personally delivered back to participants. Secondly, more rigorous field staff training in informed consent and study procedures with ongoing monitoring and supervision from researchers. Thirdly, increased earlier involvement of residents in research protocol development through study advisory groups. Additional strategies include deeper involvement of Community Advisory Groups and more focused dissemination of research results to specific audiences. In general, there is a need to identify strategies for increased accountability of researchers and participatory governance through involvement of the experimental public in all aspects of longitudinal public health research as part of the ethics in practice and democratization of science.

## Lay summary

This paper is an analysis of the views residents, service providers, and local and foreign researchers had about being involved in health research in one study area over a long period of time. It is important to understand how long term health research over long periods of time in the same population affects those involved. In this study we recorded the views of residents, service providers, and local and foreign researchers involved in health research in 31 villages in an under-developed rural area of South Africa strongly affected by the legacy of apartheid. There are some signs of development with better access to schooling, water, electricity, and shops. However, employment remains low.

Research in this study area started in 1992 to generate health and population data to inform decentralized district health systems development, policy, and planning. Health and socio-demographic information about the entire population of 120,000 people is updated annually. More recently, other studies such as testing of health service interventions have been carried out in the same study area. More effort has been put into involving research participants in research, and trying to see that they get fair benefit.

To this end we held group discussions and had individual interviews with residents, local leaders and service providers. We also asked for written reflections from researchers. The table below shows who we gathered information from:

**Table:** 

	**Individual interviews**	**Group discussions**	**Written reflections**
Residents	24	56 participants in 8 groups	
Service providers and village leaders	11	45 participants in 5 groups	
Researchers			11

The topics that we were interested in, determined as important through analysis of ethnographic fieldnotes, were:

- Informed consent, for example, why participants agree to participate in research even when they don't really understand what the research will involve
- Feeding back personal results from medical screening tests to each individual research participant.

Our results showed that health research participants needed to agree to sign multiple consent forms in order to be included in the research. We found that residents often did not understand the research. We learnt that we need to put more effort and time into training of our fieldworkers so that they fully understand the research project. Standardized training and clear guidelines for researchers about how to train and monitor fieldworkers are needed.

Participants were clear that individual results from screening tests should be delivered personally or at the time of doing the test. Researchers agreed that this was important, and that they needed to plan how to do, and pay for, this activity and include these costs as an integral part of the study budget. We also learnt that we need to think more about our employment strategies—for example, employing female fieldworkers to interview females if sensitive issues are discussed.

All participants said that activities to encourage earlier involvement and widening participation of local residents throughout the research process might prevent some of the problems that arise during research, such as rumors regarding the reasons for collection of blood samples, and consequent high refusal rates. These may help to ensure that researchers are accountable, and that residents receive full benefit from research.

## Introduction

Research conducted in health and demographic surveillance systems (HDSSs), aims to provide information that allows health policy makers and planners to deliver better health services for their populations (INDEPTH, 2012). These longitudinal centers are mostly in resource poor areas, and it is important to ensure that fair benefit of the research is considered at the local level. Public engagement activities in these centers build partnerships with local residents and service providers and support the ethical conduct of research in the field (Participants in the 2001 Conference on ethical aspects of research in developing countries, 2002; Tindana et al., 2007; Lairumbi et al., 2011; Allotey et al., 2014;Simwinga et al., 2018).

Guillemin and Gillam (2004) have suggested that ethics in practice (dealing with situations occurring during field research), needs consideration alongside procedural ethics (theory and regulatory board requirements). These situations can be called “ethically important moments” (Guillemin and Gillam, 2004: p.266), and involve “critical reflection both on the kind of knowledge produced from research and how that knowledge is generated” (Guillemin and Gillam, 2004: p. 274). Researchers working in African HDSS sites have pointed out that consideration of different cultural and social world views between participants and themselves is crucial (Duombo, 2005; Molyneux and Bull, 2013). Actions taken to alleviate these situations can lead to more nuanced and enlightened ethical theory (Guillemin and Gillam, 2004). Geissler and Molyneux (2011) utilize the term “ethos” of medical research to distinguish this type of socio-political approach to ethics in practice, which draws on sociology and anthropology in relation to a contextual approach and reflexivity in the field.

Part of ethics in practice is the important issue of fair benefit to research participants. The challenge of what is fair benefit from research has received increasing attention. The International Ethical Guidelines for Biomedical Research Involving Human Subjects, with specific reference to research in resource poor countries, state that “Before instituting a plan to undertake research in a population or community in low-resource settings, the sponsor, researchers, and relevant public health authority must ensure that the research is responsive to the health needs or priorities of the communities or populations where the research will be conducted … and … also make every effort, in cooperation with government and other relevant stakeholders, to make available as soon as possible any intervention or product developed, and knowledge generated, for the population or community in which the research is carried out” (CIOMS, 2016: p. 3). In their systematic review of nine African and seven international ethics guidelines, Lairumbi et al. (2011) found that half of the guidelines specifically discussed benefits to participants, communities and to society in general, both during and after research studies. There was considerable variation between the guidelines regarding how much responsibility researchers should have for giving benefit, as well as what these benefits might be. While there have been gains in developing ethical guidelines for health research in resource poor areas, this lack of consensus could result in different interpretations and practices regarding ensuring fair benefit from research (Nuffield Council on Bioethics, 2002; Participants in the 2001 Conference on ethical aspects of research in developing countries, 2002; Lairumbi et al., 2011; Molyneux et al., 2012).

Feeding back biomedical results that might have an impact on the health needs of individual research participants is a controversial topic that can be included in ethics in practice. Giving back results is part of the ethical imperatives of respect for person, reciprocity, beneficence, and justice (Shalowitz and Miller, 2005; Bledsoe et al., 2012), and can foster a positive attitude toward health research. Those against giving individual results argue that specimens should be given for the good of science and mankind and results might cause harm if they have not been validated, or tracking has not been adequate and the wrong result is returned (Bledsoe et al., 2012). However, in their review of articles published prior to 2005, Shalowitz and Miller (2005) found that there were very few reports of such harm, and most individuals found their test results beneficial. There is also a concern that giving back individual biomedical results might lead to “therapeutic misconception” (Appelbaum et al., 1987). This term alludes to participant's possible confusion between research and medical care and has been documented (Molyneux et al., 2005; Tekola et al., 2009). There may also be difficulties in deciding what is a “clinically relevant” result and whether only results that indicate a condition for which care can be locally obtained be returned (Murphy et al., 2008). There is an additional concern regarding cost, as giving back of individual results adds to project budgets (Bledsoe et al., 2012).

International public health research has been viewed as being carried out on “experimental publics” (Kelly et al., 2016; Montgomery and Pool, 2017; Twine et al., 2017). This term has been applied in recent public health literature to the research population in clinical trials or in this case in a health surveillance study area. The term is used as the research participants are defined by the research design and do not form a community with administrative and geographical boundaries for other purposes. In longitudinal health surveillance sites, there are regular, often more than annual updates of individual and household demographic data, Geographical Information System maps of villages, and specific smaller, nested research studies (Ye et al., 2012). Ethics in practice when working with experimental publics in these settings is particularly critical, so that vital processes of research governance which consider and include the participation and views of local residents are routinized (Nuffield Council on Bioethics, 2002; Kamuya et al., 2013;MacQueen et al., 2015).

In their work in rural Kenya and South Africa, Molyneux et al. (2009) emphasized that the relationships with fieldworkers who are locally recruited are ongoing before, during and after the research are a factor in ethics in practice. Given that in most HDSSs, there may be inequities between the researchers and locals, Emmanuel et al. suggest that considerable attention needs to be given to finding avenues to create collaborative partnerships between these parties. These partnerships allow for discussion and resolution of dilemmas, in a manner that allows different points of view to be heard, and compromises to be negotiated (Emmanuel et al., 2004).

Key to partnerships between the researcher and participants is the relationship between the field worker and the participant (Molyneux et al., 2013; Kamuya et al., 2015), which starts with informed consent. While individual informed consent is seen as a prerequisite in procedural ethical reviews, it has complexities in execution. These include how field workers understand the research processes, how they explain the methodology, how household dynamics play themselves out, local cultural beliefs, how the participants understand the information, what information is included and how the final decision is made, communicated and influenced (Tekola et al., 2009; Kamuya et al., 2015). Matters influencing the final decision can include attributes of the field worker such as whether he/she is known to the participant, age or gender disparities between the fieldworker and the participant, the real or perceived benefits from participating in the study and the level of trust placed in the researchers/research institution. In poorly resourced settings, with few opportunities for health care, decisions to participate in research may be taken in the hope that despite being informed otherwise, care might be given (Molyneux et al., 2005).

Increasingly, public engagement and participation in research is being called for at all stages of the research process, from design, through fieldwork planning, and implementation, to monitoring and analysis and distribution of results in guidelines on good fieldwork practice (South African Department of Health, 2007; UNAIDS/WHO, 2007; HPTN, 2009; UK National Institute for Health Research, 2014). Literature on public participation in science recognizes that data collection is dependent on the willingness of people to not only participate in research by answering questions and giving of their time but also sharing their local expertise and knowledge (Fortmann, 2014). Public participation in science, especially in research governance is related to civic science (Bäckstrand, 2003; Levine, 2011) and the idea that science, and health, are public goods. The notion of access to health care as a human right and as such a public good, is upheld both by the UN Universal Declaration of Human Rights—Article 25 (United Nations, 1948)—and in three sections of the South African Constitution (South African Government, 1996). The focus of this paper is on participation of the experimental public in research governance processes and will make a contribution to the growing literature on ethics in practice (Guillemin and Gillam, 2004) in longitudinal health study areas.

## Research Design

### Setting

This study was conducted in the Agincourt Health and Socio-Demographic Surveillance System (Agincourt HDSS) study area, hosted by the MRC/Wits Rural Public Health and Health Transitions Research Unit (Agincourt) (MRC/Wits-Agincourt Unit) in the rural Bushbuckridge Municipal sub-district of Mpumalanga Province, South Africa. Established in 1992, the original aim was to contribute to developing decentralized district health systems. The area is situated in the former Mhala District of the Gazankulu “homeland” formed during the apartheid years. These areas, under self-rule but not independent, suffered limited development and poor investment in health, infrastructure and education (Niehaus et al., 2001). In 1994, South Africa held its first democratic elections, and a new democracy was born. Under this new system, over a period of time, the area was renamed Bushbuckridge. The area is situated 500 km north east of Johannesburg, and is still characterized by high unemployment, with high rates of labor migration and a legacy of the apartheid system of forced labor migration. Poor education standards persist and, although infrastructure has seen some considerable development since 1994, there are still poor roads and limited water supply (Kahn et al., 2012; Collinson et al., 2014). Annual health and socio demographic census updates have been conducted with the 116,500 people residing in 21 300 households in the 27 adjacent villages in the Agincourt HDSS since 1992. Updates include information on births, deaths, in and out migration, education and socio-economic status, family structure and various, scheduled updates on, for example, food security, and health care utilization.

Despite an increased focus on access to health care post-*apartheid*, access remains inequitable in South Africa (Harris et al., 2014). Findings from the Agincourt HDSS and its nested studies, particularly those that indicate rapid health, social, and demographic transitions, contribute to health policy and planning (Tollman, 2008). The objectives of the MRC/Wits-Agincourt Unit have expanded to include reasons for, and dynamics of, these transitions, deepening observational work through cohort studies. The unit also conducts intervention studies with cross-site collaboration, and produces public access datasets, with the goal of mounting more effective public health, public sector and social responses (Kahn et al., 2012).

**Figure 1:**
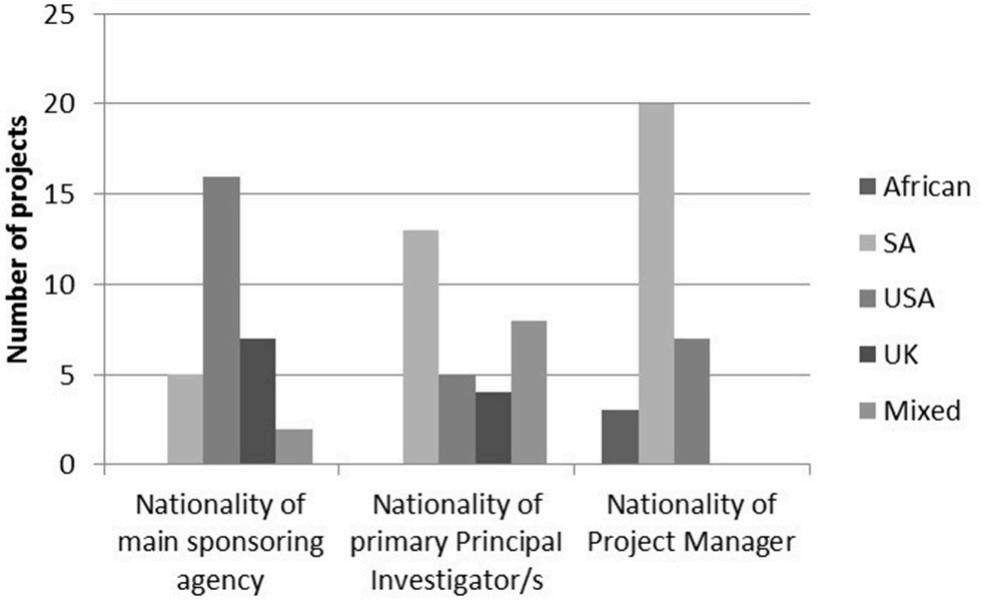
Nationalities of main sponsoring agency, primary principal investigator/s, and project managers in 2018.

The work of the MRC/Wits-Agincourt Unit is collaborative, international and the boundaries of the work are global. It is one of the few HDSS sites worldwide that is led by an academic institution based in the host country. Other research studies, including trials, observational, and intervention studies, run by local and international collaborators have been nested in the Agincourt HDSS using the HDSS dataset for sampling (Gómez-Olivé et al., 2013; Thorogood et al., 2014; Pettifor et al., 2016; Gaziano et al., 2017). Although most projects are still internationally sponsored, there are growing numbers of South African principal investigators, and South African and African project managers working in the site. In 2018, there were 30 nested studies at various stages of which nine were led by international collaborators, 13 South African led and eight jointly led (Figure 1).

All projects based in the MRC/Wits-Agincourt HDSS can be classified as community-based, and public engagement is intrinsic to such research. A Public Engagement Office (PEO) was formally started in 2004, to formalize and expand previous public engagement activities. RT leads this office. The PEO works with Principal Investigators and project managers of studies, keeping investigators alert to ethics in practice issues. There is a Community Advisory Group (CAG) consisting of one person elected by the Community Development Forum (CDF) of each village that meets monthly. Smaller study advisory groups, comprising eight randomly selected CAG members are formed for most nested studies. There are village-based meetings and targeted briefings with traditional and civic village leaders, local, district, and provincial governmental and relevant non-governmental service providers, before a study commences to discuss the upcoming project, and at its conclusion to disseminate results (Twine et al., 2017).

### Study Procedures

This is a case study using multiple qualitative methods that included semi-structured individual, focus group and natural group interviews, ethnographic field notes, and critical incident scenarios (Crisp et al., 2005). The semi-structured individual, natural group, and focus group interviews were conducted with village residents, local leaders, and service providers all from within the study area. These interviews explored their experiences of being involved in the activities of the longitudinal research site. Interview guides were field-tested with the Community Advisory Group. Natural group interviews are group discussions that occur with people forming an existing group so all the participants know each other. Generally, they are based round a shared interest (Beckerleg et al., 1997; Green and Thorogood, 2009). Group interviews with resident groups were natural group interviews but the group interviews with village leaders and home-based carers were focus group interviews.

To recruit village residents, two villages with diverse characteristics were chosen—one far from and one close to the MRC/Wits-Agincourt Unit offices, one with a large and one a small population, and one with a higher and one a lower average household socio-economic status. A table outlining how many participants were needed from each village, ensuring gender and spread across three age groups (18–24, 25–49, and 50+ years). The fieldworkers recruited door-to-door until there were 24 eligible participants. None of the participants were known to the fieldworkers previously. Eight natural group interviews were also conducted with an average of ten participants in each group. Natural groups were made up of: older men who were assistants to the village chief and a group of cattle herders; younger men in a soccer team and in a traditional dance team; older women attending church or who drank tea together; younger women from a church group or a traditional dance team (Table 1). Interviews were conducted by two local, Shangaan speaking fieldworkers in 2016, at participant's homes or other locations of their choosing, and no one apart from the participants and the fieldworkers were present. To avoid socially desirable responses, the interviewers were trained to encourage critical views by explaining that only through these can practice be improved. The reasons for the research were also outlined in the consent form.

**Table 1:** Research participants living within the study area—“residents.”

	**Individual interviews**	**Group interviews**
Local village residents	24	56 participants in 8 groups
Service providers and village leaders resident in the area	11	45 participants in 5 groups

A purposive sample of 56 local leaders and service providers was selected from individuals working within organizations involved in governance or service provision at the village and sub-district level, and who were also resident in the study area. Some of these participants knew RT prior to the interviews. Recruitment and logistical arrangements were telephonic. There were 45 participants in the focus group interviews and 11 in individual interviews. Two representatives from village leadership from each of the 23 villages that had been involved in the HDSS for over 10 years, participated in four focus group interviews of between eight to eleven participants and the managers of eight home-based care organizations participated in another focus group interview (Table 1). Representatives from the traditional councils and municipalities, clinic, and education managers were all interviewed individually. The participants were aged between 25 and 70 years, and were balanced by gender. RT conducted these interviews and the natural group interviews along with a fieldworker in 2015/16. Interviews were undertaken in a venue in the village chosen by the participants, and no-one was present aside from the researchers and the participants.

Data from residents' interviews were analyzed by RT in 2018 focusing on ethics in practice. The emergent themes were informed consent, collection of body tissue samples, confidentiality, adverse events, referral vs. health care provision, end of study withdrawal and benefits such as the HDSS providing employment.

RT took field notes on ethics in practice incidents in the study area during 2015–2017. The purpose of these field notes was to capture and reflect through “thick description” (Geertz, 1973) on “ethically important moments” (Guillemin and Gillam, 2004: p. 266). In 2018, three critical incident scenarios (Crisp et al., 2005) were selected from the field notes depicting situations illustrating the ethics in practice issues that local residents had raised. They were on informed consent, giving back of individual screening results and adverse events. In this paper two are being used. All three scenarios were sent electronically to 10 purposively selected researchers who had been involved in nested studies in the Agincourt HDSS. The criteria for their selection were that they had worked within the study area on a nested study within the last 3 years and equal representation was given to researchers from South Africa and external to South Africa. The ten individuals included: principal investigators, research managers, project site managers, and project coordinators (Table 2). Any researcher who was employed by the HDSS was excluded; this involved 4 men and 1 woman. The researchers who met the criteria included 8 female and 2 male researchers. Gender was not a consideration in the selection of the sample, rather the focus was on having carried out research in the study area within the time period and not being an employee. All the researchers responded with reflections and comments. The case studies were anonymised so that the study was not identified, and as the researchers were sent the scenarios electronically and replied individually, there was no known sharing of reflections.

**Table 2:** Senior researchers and senior field staff responding to critical incidents—“researchers.”

	**Permanent resident or citizen in South Africa**	**International**	**Total**
Senior researchers—principal investigators and project managers	1	4	5
Senior field staff—project site managers and project coordinators	4	1	5
Total	5	5	10

Participants were given 2 weeks to reflect on the scenarios and respond to two questions: “Describe how you would have taken action (if any) if you were in the research team involved” and “What issues does this scenario raise regarding ethics in practice (ethical issues that arise during fieldwork)?”

### Analysis

All interviews were digitally recorded with the exception of one interview where the participant refused and field notes were taken. The recordings were translated and transcribed from Shangaan into English by the local fieldworkers. Transcripts were not returned to participants for comment as they had been transcribed directly from Shangaan recordings into English. Selected transcripts and questionnaires were read in full by RT and GH independently in order to identify emergent themes for the initial coding, which was both deductive following the topic guide and inductive in terms of emergent themes within the topics and in addition to the topics. QSR NVivo software (version 10) was used for the coding of interviews with residents. RT undertook manual thematic analysis for the data from researchers.

### Ethics Approval and Consent

Ethical clearance was obtained from the University of the Witwatersrand's Human Research Ethics Committee (Medical) (Certificate numbers M140361 and M140737) and permission for interviewing service providers obtained from both the Mpumalanga Provincial Departments of Health and Education Research Offices. Written informed consent was gained from all participants prior to data collection.

## Results

### Informed Consent

All interviewed residents had been participants in the annual HDSS census update and in various nested research projects, and reflected on their experiences of informed consent. Some residents mentioned that the process had been clear and that they had known what they were agreeing to, but there were instances where a resident, or a family member who had been approached to be a study participant, had not understood fully what agreeing to participate in the study involved.

“If you don't understand, the field workers give you a chance to say that. They say that participating is voluntarily. You are allowed to say no. Even during the interview, they allow you to stop if you are not comfortable with their research.” *Middle aged man 3, village 1*

“The problem is that they don't say what is going to happen at the research laboratory. My grandmother was supposed to know what will happen to her. She needed to have more information.” *Young woman 2, village 1*

Although some residents said that they had understood the reasons for the research, others said they had not. Residents also spoke about instances when they had asked the field workers questions about the reasons for the research, and the field workers themselves did not know.

“I don't have a problem with these questions as the one who came explained everything. They were checking whether we are eating modern food only and not cultural food. That's why they are asking all these questions.” *Older man 8, NGI village 2*

“I don't want to be asked questions about food as they won't give me money to buy food afterwards. The problem is that they don't tell us why they are asking these questions. All they say is that they are working.” *Older man 4, NGI village 2*

The majority of the residents described a high level of trust in the field workers employed in the MRC/Wits-Agincourt Unit, referred to as “Wits” locally. They said that the field workers were well trained and respectful.

“They introduce themselves and they ask for your time. Though the research questions are not good, the field workers are respectful.” *Older man 4*

“When they approach your gate they are smiling, they greet you and they will introduce themselves, telling you where they come from. They will ask for your permission to work and afterwards they will say thank you.” *Older man 1, NGI village 2*

“If I have problems and I don't have someone to share my problems with, I can share with Wits people, particularly when that study is related to my problems.” *Middle aged woman I, village 2*

The signing of consent forms without understanding the implications was raised as an issue for older participants. Owing to a high level of trust and respect for the field workers, residents thought that older people sometimes agreed to answer the questions even if they did not fully understand the reasons for the study.

“Yes we understand most of the information on the informed consent. Some read it and sign with understanding. But with old people I think they don't understand everything it would be better if you read it when there is a relative there who can understand what you are saying. Old people will agree to anything as a sign of respect although they didn't understand. I think your field workers need to take their time in the field.” *Middle aged woman 1, village 2*

Residents did talk about particular instances where they felt uncomfortable divulging confidential information to young field workers on sensitive issues such as the nested research studies on aging which have sexual behavior questionnaires that include topics such as frequency of having sex, multiple sexual partners, and contraception. Disclosing details about intimate sexual behavior with a young person was considered inappropriate and there were some doubts about confidentiality.

“In our culture we were taught that you talk about sex in your bedroom with your partner. But with Wits, they send a young girl to an old person to ask those questions. We don't know whether they are going to keep the secrets as we don't know them. We used to lie.” *Young woman 1, village 1*

As informed consent was a key concern to interviewees, the critical incident scenario in Box 1, summarized from RT's field notes was sent to 10 researchers.

Box 1 **Scenario on informed consent**

The recruitment of young women for a study involved consenting for HIV testing. In this case, the young woman was 13 years old and lived with her maternal grandmother. Her father lived elsewhere and her mother died 9 years previously. As per approved procedures, the father was called by cell-phone to obtain consent for the caregiver (grandmother) to provide consent for the young woman's participation in the study. The field worker did not speak directly to the father, but allowed the grandmother to conduct the conversation—and the grandmother did not inform him of the HIV testing component of study enrolment. This constituted a protocol violation as the field worker should have personally had this discussion with the father. The father and grandmother and the young woman consented. The young woman was found to be HIV positive during testing and she told her grandmother the result of the test. The father contacted the study team, angry that his daughter was tested without his permission. It appears that the young woman was infected perinatally and that her father had not informed her, nor her grandmother of her status.

Researchers' responses to this situation were that it is a complicated situation that has implications for the participant and the family, the study itself and for future nested studies in the longitudinal study area.

“Firstly, there is need to protect the study from possible withdrawal by the participant and other participants which would affect other studies of the Unit. Secondly there is need to protect the life of the young girl by ensuring that she gets all the necessary clinical and family care. Thirdly there is need to protect the family from possible conflicts and disintegration.” *Senior Field Staff 2*

Researchers talked about field workers, despite being trained, being under pressure and taking shortcuts in order to meet targets.

“This brings up two issues. The first is the field worker violating protocol. Unfortunately, this happens despite careful training and a detailed protocol. Situations arise that are not straightforward (this situation is unlikely something the fieldwork team had discussed or planned for) and field workers do not always make the right choice and often do not ask their supervisors for advice. Field workers need to be trained to ALWAYS ask for advice and direction when in doubt of proper procedure. This kind of scenario requires further discussion and training.” *Senior Research Staff 3*

Researchers, like the residents, also mentioned that older participants might have more difficulty understanding research processes.

“The field worker was supposed to talk to the father directly and not via the grandmother. The field worker had more information about the study and HIV testing compared to the grandmother. The grandmother did not know the major issues surrounding HIV/AIDS.” *Senior Field Staff 2*

Researchers spoke about the importance of field worker training and quality assurance procedures being in place to ensure that proper informed consent practices are followed.

“The training for the field workers needs to revised and reinforced and maybe the research manager should consider whether there are adequate on-going quality checks”* Senior Researcher 5*

### Giving Individual Results From Clinical Screening Conducted as Part of a Study

Increasing numbers of studies in the site include some form of clinical screening in addition to interviews. For example, this can be measuring blood pressure, taking venous blood for glucose levels, dry blood spots for HIV testing or collecting urine samples. Residents liked having their individual results from these tests immediately.

“Researchers came to my house and checked us, blood was taken by pricking our fingers and results were given at the same time. They also checked our blood pressure…. this helped me …as I was given the results at the same time. I was happy as they came to our home and checked the whole family including the elders. We were all given the results. I remember my mother's blood pressure was high as she was angry that morning. She was told and given a referral letter to the clinic and she came back home with treatment.” *Young man 1, NGI village 1*

However, there were many instances where residents talked about either themselves or people they knew who had had blood taken and did not receive their results.

“But there is a participant who told me…….they had taken a lot of blood and this worried him a lot because he didn't get any results after they took his blood.” *Headman 2*

In the past, for tests without immediate results participants were sometimes referred to the clinics to get their results. Residents felt that if the researchers could arrange to collect tissue samples at participants' homes, or transport participants to the research laboratory to collect samples, results should be delivered to them personally at home. The clinic managers also expressed challenges with giving research screening results as there were delays in getting the research results to the clinics, and participants became irritated.

“You cannot take blood from one person but not give results. Then you come again and you want to collect more blood for another study. Where is the first blood? Where did you send it? People need their own results and not as a group. My child's [nasal] mucus was taken, but there are no results. I think that is wrong…. Bring back your findings. If you can do so, people will be interested to participate. That's my request.” *Young woman 2, village 1*

“A challenge I had was that there are those who are being tested for HIV at their homes and being given stickers to come to the clinic for the results. Someone in the clinic had to check for their results in the computer. The results were not available even though it was after quite a long period. That can lead people to not accept field workers the next time because they have had a bad experience.” *Clinic operations manager 3*

Residents, particularly service providers and local leaders, were clear that more consultation earlier in the research process would be helpful to everyone.

“We need to consult with the community. Then the community will come up with ideas of how exactly we can improve.” *Participant 7, FGI4 CDF*

Given that getting individual screening results was an important concern in almost all the interviews, the following scenario in Box 2, based on field notes about an actual critical incident, was sent to 10 researchers for their views.

Box 2 **Scenario on giving back results.**

An information sheet and informed consent form was sent to the Public Engagement Office for review. Participants were being asked to give a blood sample for HIV testing, but there was no mention in the informed consent of how the participants were going to be given the HIV test results. Upon follow up with the Principal Investigators, it was confirmed that there was no plan for reporting back individual HIV results to participants, and no budget for this. It emerged that the US partner in the study had previously requested more money from the budget for study costs in the US, and this request had been accommodated by the investigating team.

All the 10 researchers wrote that it was ethically important to give participants back results from screening tests. Some acknowledged that although research may only have policy impact later on, more immediate benefit to participants is important and a right.

“If you are going to require them to give you their time and physical bodies for your research then you must show respect by letting them know the results of the test you are conducting, particularly if it is a test that is of high burden in their community and could save their life and the lives of other people.” *Senior Research Staff 3*

Researchers also wrote that giving back of results would assist future studies in the longitudinal health research area, by helping to maintain trust.

“We have to do this to prevent refusals and the researchers must not take advantage of people participating in their study….if they [participants] think that they have been used but didn't get their results, they will refuse when other studies similar to that one come.” *Senior Field Staff 3*

“It also raises an issue of partnership ethics. The US partner is weighing their needs higher than the local implementing partner which is also a violation of respect for persons. Given the local budget is running the project I would emphasize the US partner needs to be more accommodating, as without the local buy in, there is no study.” *Senior Research Staff 3*

Researchers problematized the giving back individual results as part of research activities, but were clear it was sensitive and required planning, consultation and funding. A researcher noted that there is a tension between availability of funds and costs of giving back individual screening results, and that international researchers needed to be mindful of fair benefit and researcher accountability to the experimental public.

“….giving back the results …. must be done carefully. The research participants must consent and suggest where he/she would be comfortable to get the results. Some would not be happy to have their results at the clinic and that needs to be considered.” *Senior Field Staff 2*

“Research should be adequately funded, allowing for treating the participants with consideration and dignity. Maybe, in future, this should be considered earlier in the development process.” *Senior Research Staff 5*

Researchers also mentioned the importance of thinking about giving back of results during the project planning phase, and including local researchers and residents in project planning.

“Why was reporting of individual results not a priority during proposal and budget development? What did the study team plan to do when they got the HIV results?” *Senior Field Staff 4*

## Discussion and Conclusion

The findings from this study using multiple qualitative methods have implications for widening participation of the experimental public as part of study processes in longitudinal health research sites. Issues that arose relating to informed consent and giving of individual results from screening tests are discussed.

Public health research studies often involve complicated field work processes, with multiple informed consent sheets. It is clear from the results that the resident interviewees felt that sometimes neither participants or field workers fully understood study activities, nor the reasons for the research itself. This was reported as being more of an issue with older people. Age differences between participants and field workers was important when older participants were reported as being reluctant to answer questions on their sexual behavior to young field workers, or those of a different gender to themselves.

Residents also reported that, especially but not only for older people, a high level of trust in and respect for field workers influenced participants to sign consent forms despite not understanding the implications. Researchers said that if information in the consent was misunderstood, or not understood, and unrealistic expectations raised, there would be implications for the participant, his/her family, the study itself as well as for future studies in the study area.

The results in this paper reinforce previous findings that informed consent is often complex and requires careful attention. Molyneux et al. (2005) also highlight that the decision to sign an informed consent may be made because of a high level of trust in the field worker and the research institution, or because of real or perceived benefits from the study. Kamuya et al. (2015) and Tekola et al. (2009) discuss the complexities of gaining informed consent in research studies, noting the importance of how and what information is presented, and that cultural issues affect the decision to sign consent. Field worker training and support can mitigate ethical issues that occur in the field (Tekola et al., 2009; Kamuya et al., 2015) and it is clear that training at the onset of a study needs to be followed up with frequent monitoring and supervision of the field workers on the taking of informed consent. Calls for standardized training for field workers have been made at a workshop in 2015 involving nine African longitudinal health research institutions in Kombe (2015).

Cultural considerations regarding older people's lack of trust in younger fieldworkers, or of younger fieldworkers contravening cultural practices through having to ask sensitive questions to their elders have also been discussed in relation to informed consent in other HDSS study areas (Tekola et al., 2009; Kamuya et al., 2015). The older population in this study area understands research to a lesser extent than the fieldworkers owing to disparities in access to education during the apartheid area. In 2010, one study in the HDSS found that of 5,056 people aged 50 years and over, over 55% had no formal education and 24% had six or less years of education (Ameh et al., 2014). Owing to cultural changes, younger fieldworkers may respect their elders less than in the past (Stadler, 2003). This may lead to elders being submissive, or untruthful in their responses. A current related dilemma in this research setting, is that younger fieldworkers, owing to greater access to post-secondary education post-apartheid, are more likely to be appointed as fieldworkers than applicants who are older. This is considered a benefit by the population in the area, as youth unemployment is extremely high. These fieldworkers are also more likely to understand research and be able to use technology which is vital as data collection has moved from being paper-based to electronic.

Participants appreciated receiving individual results at the time of doing the screening tests, but were clear that results from samples sent off for testing should be delivered personally, or given at the time of doing the test, whether positive or negative. Researchers agreed that there was an ethical imperative to give participants their results, both immediately from screening tests and for those that were sent away for analysis, were positive and clinically relevant and for which treatment was available locally. This would benefit individuals, and future research studies would also benefit as participants would feel that their dignity and interests was being respected and would be more willing to participate in further studies. Researchers wrote that giving individual results required careful planning and resourcing, needed to be included from the proposal development stage, and that this consideration of fair benefit may require budgetary adjustments.

Supporting the findings from Bledsoe et al. (2012), no adverse events were reported by participants regarding receiving individual screening test results, and giving individual results seemed to create a positive attitude toward research, and was seen as a fair benefit from the research (Shalowitz and Miller, 2005; CIOMS, 2016). Provision of individual screening results as part of public health research in general rather than specifically in longitudinal settings is only mentioned in one guideline ICH-GCP (1996) in Lairumbi et al.'s (2011) review of research ethics guidelines. It is clear from this paper that participants view this as a real benefit. In countries such as South Africa, where there is primary health care free for many conditions, there may be less risk of therapeutic misconceptions (Appelbaum et al., 1987; Molyneux et al., 2005) when giving individual test results.

Currently in this HDSS, consultation with the PEO and the CAG often only occurs after proposals have been written, funded and ethical approvals obtained. Widening participation through mechanisms for consultation with residents and researchers regarding activities in a longitudinal health study area could assist in guiding decisions around governance in all these research activities, in order to enhance both accountability of researchers and fair benefit (Bäckstrand, 2003; Emmanuel et al., 2004; Levine, 2011; Kamuya et al., 2013; Molyneux and Bull, 2013,;Simwinga et al., 2018).

### Implications for Practice in Longitudinal Health Study Areas

These issues are not unique to this rural, South African setting and there are implications for other longitudinal health study areas globally. There is a need to identify strategies and mechanisms to ensure increased accountability of researchers and stronger participatory governance through involvement of the experimental public in all aspects of longitudinal public health research as part of ethics in practice. From these findings, two strategies have been identified by researchers and residents: improved field worker training and ongoing supervision during data collection, and increased involvement of residents in protocol development, data collection and dissemination.

Development of accredited training modules on informed consent and other ethics in practice for field workers is one strategy to address some of the informed consent issues. More time needs to be budgeted for training, so that research teams can be certain that fieldworkers understand the reasons for the research and the fieldwork processes. Understanding findings from prior research in the study area will allow fieldworkers to better understand the reasons for the research and possibly allow for more targeted dissemination of findings to participants. In areas where research is conducted in collaboration with external principal investigators and research managers, adequate orientation on public engagement, field operations, and ongoing supervision requirements for fieldwork is needed. In this HDSS, there are frequent meetings between on-site research managers and field teams. One possible way forward could be to have a monthly ethics in practice forum for fieldworkers and research staff to reflect on ethical dilemmas encountered in the field. These are essential to supporting fieldworkers, and allow for team discussion around dilemmas that may arise. Additionally, monthly meetings between research managers of different nested projects to discuss fieldwork issues enhances their ability to manage fieldwork. Clear guidelines for principal investigators and research managers outlining requirements for protocols, management of ethical issues, public participation, training, and monitoring of fieldworkers also need to be in place and accessible.

One strategy for widening participation is a CAG (Lairumbi et al., 2011; Simwinga et al., 2018). CAG members need adequate training and a constitution that is upheld, for example regarding length of terms of office. With the growth of nested research studies in this HDSS, monthly CAG monthly meetings cannot engage with the detail and governance of each project so Study Advisory Groups were established to advise on information sheets, review topic guides and advise during data collection and dissemination.

Other strategies to widen participation in longitudinal health research areas could include more considered approaches to recruitment and deployment of fieldworkers, ensuring for example that female fieldworkers interview female participants if there are sensitive issues to be discussed, more focused dissemination of research results to specific audiences, monitoring of reasons for refusal to participate and suggestion boxes in the study area. A number of these strategies have been implemented in the study area already, and more strategies to widen participation are planned, including regular focus groups with individuals and service providers around their experience of living and working in this study area. A key lesson learnt during implementation of strategies to widen participation is that it is not possible to include all residents in the study area, and champions are important, but representation needs careful consideration. Public participation in research is not static, and continued assessment of existing strategies is required, consultation and development of new relationships should be ongoing (Lavery et al., 2010).

This paper builds on and extends previous work on ethics in practice in longitudinal health research areas. It highlights the importance of widening the participation of residents who form the experimental public in research governance mechanisms in these settings in order to ensure the longevity of these institutions. Widening participation is intrinsic to the democratization of science as a public good (Bäckstrand, 2003; Levine, 2011) and can enhance both the lives of research participants and the quality of the research.

## Ethics Statement

Ethical clearance was obtained from the University of the Witwatersrand's Human Research Ethics Committee (HREC) (Medical) (Certificate numbers M140361 and M140737) and permission for interviewing service providers obtained from both the Mpumalanga Provincial Departments of Health and Education Research Offices. Written informed consent was gained from all participants prior to data collection.

## Author Contributions

RT, GL, and KK planned the study. RT implemented the study and RT conducted the analysis and linked the findings to the literature, supported by GL. KK provided critical revision of the manuscript.

### Conflict of Interest Statement

RT and KK both work for the MRC/Wits-Agincourt Unit in which this study was undertaken. The remaining author declares that the research was conducted in the absence of any commercial or financial relationships that could be construed as a potential conflict of interest.
